# Fermentation Quality and Additives: A Case of Rice Straw Silage

**DOI:** 10.1155/2016/7985167

**Published:** 2016-06-27

**Authors:** Yusuff Oladosu, Mohd Y. Rafii, Norhani Abdullah, Usman Magaji, Ghazali Hussin, Asfaliza Ramli, Gous Miah

**Affiliations:** ^1^Institute of Tropical Agriculture, Universiti Putra Malaysia (UPM), 43400 Serdang, Selangor, Malaysia; ^2^Department of Crop Science, Faculty of Agriculture, Universiti Putra Malaysia (UPM), 43400 Serdang, Selangor, Malaysia; ^3^Department of Biochemistry, Faculty of Biotechnology and Biomolecular Science, Universiti Putra Malaysia (UPM), 43400 Serdang, Selangor, Malaysia; ^4^Strategic Livestock Research Centre, Malaysian Agriculture Research and Development Institute (MARDI), 43400 Serdang, Selangor, Malaysia; ^5^Rice Research Center, Malaysian Agriculture Research and Development Institute (MARDI), 43400 Serdang, Selangor, Malaysia

## Abstract

Rice cultivation generates large amount of crop residues of which only 20% are utilized for industrial and domestic purposes. In most developing countries especially southeast Asia, rice straw is used as part of feeding ingredients for the ruminants. However, due to its low protein content and high level of lignin and silica, there is limitation to its digestibility and nutritional value. To utilize this crop residue judiciously, there is a need for improvement of its nutritive value to promote its utilization through ensiling. Understanding the fundamental principle of ensiling is a prerequisite for successful silage product. Prominent factors influencing quality of silage product include water soluble carbohydrates, natural microbial population, and harvesting conditions of the forage. Additives are used to control the fermentation processes to enhance nutrient recovery and improve silage stability. This review emphasizes some practical aspects of silage processing and the use of additives for improvement of fermentation quality of rice straw.

## 1. Introduction

Rice generates a relatively large amount of crop residues known as straw. These residues are the leftover vegetative parts after harvesting the grains. Rice straw is made up of panicle rachis, leaf blades, leaf sheath, and the stem. The average ratio of the rice grain to rice straw is 1 : 1.25 [1]. Only about 20% of these straws are used for industrial (e.g., ethanol, paper, and fertilizer) and domestic (e.g., fodder) purposes [2]. Most of the remaining straws can be removed from the field, left undisturbed to serve as mulch, ploughed into the ground to add nutrients to the soil, or burnt. Burning of rice straw is the fastest mode of straw disposal but causes environmental pollution by increasing the amount of greenhouse gas in the air [3]. Rice straw is used as part of the nutritional requirements of ruminant animals in most rice producing countries [4]. However, low protein content, possession of phenolic properties, and high level of silica and lignin are the primary limiting factors in rice straw digestibility in ruminant animals [5].

These constraints have to be addressed before rice straw can be considered as a potential ingredient in ruminant diet [6]. In the past, many attempts have been made to increase the digestibility and utilization of the agricultural residues. One of them is biological treatment (white rot fungi and enzyme). Some, however, focus on physical (grounding, steaming, and pelleting) and chemical treatments (urea, ammonia (NH_3_), sodium hydroxide (NaOH), and lime) to improve the feeding values of rice straw. Of all the available methods of improving rice straw, biological and chemical treatments are mostly used [5]. These treatments involve the use of urea as a source of nitrogen which serves as a delignifying agent through ammoniation. Also it was reported to remove the polymerized silica from leaf sheath and leaf blade [7]. According to Sarnklong et al. [8] sodium hydroxide was regarded as the most effective treatment, but its major disadvantage in animal is that it causes faster rumen washout and heavy urination [5] which shows that these methods are still ineffective. However, there has been a great improvement in feeding value of rice straw by ensiling in recent times [9, 10]. Therefore, there is need for providing adequate knowledge on the existing ways of improving nutritive value of rice straw to allow for its efficient utilization. Successful ensiling of rice straw is difficult due to its hollow stem, low water soluble carbohydrates (WSC), and less epiphytic lactic acid bacteria (LAB) [9]. Nonetheless, improvement of the silage stability and consequent improvements in animal performance can be achieved through application of the fundamental knowledge of the process, understanding the complex factors involved in natural microbial population and harvesting conditions of the forage, enhancement of nutrient, and energy recovery [11]. This review emphasizes some practical aspects of silage process based on available information from different authors describing the purpose of using silage additives in order to improve its nutritional values and encourage lactic acid fermentation to inhibit undesirable microbes. Therefore to understand more the above information this review is made from different sources.

## 2. Abundance of Rice Straw

Global rice production has dramatically increased at an average rate of 2.5% per year in the last decade, reaching about 744.4 million tonnes in 2014 [12]. Approximately 90.49% of the global rice straw is from Asian continent; Table 1 shows the abundance of rice straw across the six continents in 2014. Asian countries have been associated with higher availability of rice straw and it was calculated that utility of these crop residues amounted to 20% of the total crop residue [2]. The assertion of Devendra and Thomas [13] that 90% of ruminant animals in tropical Asian countries are fed with rice straw might be consequential upon the abundance of rice straw in the area.

## 3. Environmental Effect of Burning Rice Straw

Rice straw burning is the cheapest, fastest, and most effective method of straw disposal. However, burning of rice straw can lead to incomplete combustion releasing a large amount of air pollutants such as volatile organic compound (VOC), carbon monoxide, fine/inhalable particles, and carcinogenic polycyclic aromatic hydrocarbons [33]. Biomass burning has been reported to be potentially poisonous [34]. Also in Japan, straw burning is linked to the cause of asthma in young children [35]. In addition, burning of agricultural residues is said to be instrumental to formation of brown cloud which affects the quality of the air, atmospheric visibility, and global climate [36]. Burning of crop straws causes loss of nutrients about 30 to 35% of phosphorus (P), 40% of nitrogen (N), 40 to 50% of sulphur (S), and 80 to 85% of potassium (K) [37]. Nevertheless, the amount of nutrient loss depends upon the method used for burning. In countries like Thailand, China, and northern India where mechanized harvesting is practiced, all the straws left on the field are burnt in situ. In this method, loss of minerals is less compared to countries like Indonesia and the Philippines where straws are heaped into piles after threshing and burnt. In the latter method, the resulting ash is not evenly distributed on the field and this results in high loss of minerals. Burning is a cost effective method for straw disposal and also helps to reduce disease that may occur due to reinfection from inoculum in the straw biomass [38]. Therefore, it hastens planting/sowing of subsequent crops. On the other hand, ploughing down straw aids spread of fungi [2]. However, use of rice straws as livestock feed is a cost effective way of reducing the environmental impact of burning and perhaps the best use of rice straw as an energy source will be achieved.

## 4. Limitations to Rice Straw Utilization 

Digestibility is the amount of nutrients absorbed from the feeds by the host animal from the disintegration and fermentation by rumen micros making nutrients available for growth, reproduction, and so forth. The forage digestibility and the ruminant's capacity to consume it are largely influenced by cell wall and its contents. Cell contents and perhaps small amount of fiber are the only digestible parts of a plant. Animals can easily digest the plant cell contents, but not their cell walls. The cell wall of rice straw is made of approximately 5.5% lignin, 40% cellulose, and 18% hemicellulose [39]. Lignin is the main component of cell walls. It is an amorphous polymer made up of different phenolic compounds. It functions as a mechanical support for binding plant fiber together. Also, it reduces the permeability of water through the xylem and thereby plays an active role in water and nutrient movements. In spite of these functions, lignin cannot be digested no matter the duration or retention in the digestive tract [40]. In addition to that, there is reduction in the amount of energy derived from straw feed because ruminant animals lack specific enzymes for cellulose digestion [41]. Rice straw has an extremely high silica content of about 15% of its dry matter [8]. Silica in the soil is absorbed and metabolized by plants and plays an important role similar to lignin [42]. Cellulose in the plant is composed of both crystalline and amorphous structures. The level of crystallinity of cellulose is believed to affect the rate of its decomposition by the cellulolytic organisms. High level of crystallinity decreases the rate of microbial degradation of cellulose [43]. Generally, rice straw contains high fiber, silica, and lignin which are slowly fermented in the rumen when compared to other forage crops. Therefore, feed intake is reduced if the ration is majorly from rice straws.

Van Soest [44] opined that feed intake is limited by the amount of fiber in the diet when cell wall content lies between 50 and 60% of forage dry matter. Also the voluntary intake is expected to be inversely related to the fiber content of the forage because further intake is limited as the slower digesting fraction becomes large in relation to the volume of the digestive tract. In the same way, particle passage is expected to decrease with increasing neutral detergent fiber (NDF) intake, particle size, coarseness of forage, and decreasing forage digestibility. Rice straw contains 3–5% crude protein; animals fed with unsupplemented straw diet only will very often lose weight. However, 8–10% of crude protein in animal feed is required for improved consumption and good growth [45]. Nutritional quality of rice straw also depends on numerous factors such as plant maturity stage (lignin content increases based on maturity stage); fertilizer level (nitrogen fertilization); soil fertility; ratio of leaf sheath, leaf blade, and stem; length of harvested straw; degree of senescence of straw during harvest; plant resistance to pests; time between harvesting and storage; and plant health and weather conditions [5]. Improvement of this valuable fodder crop is of great importance so as to create environment-friendly rather than cultural practices of burning. In the past, many attempts have been made towards increasing the utilization and digestibility of these agricultural residues, such as physical pretreatment of biomass with the purpose of increasing the available surface area and the size of pores of cellulose by grinding and milling. The result of this treatment is not satisfactory because it is often used in combination with other pretreatments such as chemicals and biological treatment in order to improve the efficiencies. However, this method is not cost effective because of financial and economic implications [46].

## 5. Silage Fermentation Process

Silage is the product formed when grass or other green fodder with sufficient moisture contents is stored anaerobically, typically in the silo after wilting, to prevent spoilage by aerobic microorganism [47]. The fundamental principles of silage process are maintenance of anaerobic conditions throughout the ensiling and rapid decline in pH value by lactic acid bacteria [48]. Historically, forage is preserved as hay (by drying) or as silage (by acidification or sterilization). Hay works well in dry climates for forage crops which dry quickly. The key principle is rapid drying to <15% moisture in order to prevent mould growth and formation of heat from aerobic bacteria. However, in situations where dry conditions are uncertain, then ensiling generally makes sense in order to preserve as much of the feed nutrient value as possible. The primary goal of making silage is to enhance fermentation by using crops with high levels of epiphytic (existing on the plant) bacteria and sugars in the crop to allow anaerobic bacteria to create acids which essentially pickles the crop to give it long-term stability. Numerous reasons for forage ensiling are convenience, cost effectiveness, weather, and crop characteristics. To achieve a good silage product, understanding the condition for ensiling process is a prerequisite. This is categorized into four phases [14, 49].

### 5.1. Aerobic Phase

The first phase is characterized by utilization of trapped oxygen during ensiling process. Chopped and packed forage in the feed storage is characterized by the presence of trapped air during ensiling. During the process forage plants continue respiring for several hours and possibly days depending upon the package [48]. During this phase, plant enzymes (proteases) are active until the trapped oxygen is exhausted. If the ensiling is poorly packed or it allows penetration of oxygen, the forage undergoes undesirable fermentation by moulds and yeast leading to breaking down protein and excessive heating. These can be controlled by chopping the forage crops into correct lengths, tightly packing them in the storage structure, excluding all the trapped air from forage mass, and immediate packing as delaying might have a detrimental effect on silage quality [50].

### 5.2. Lag Phase

This phase is characterized by depletion of available oxygen in the ensiled materials through the action of anaerobic bacteria. Silage conditions such as availability of substrate for microflora growth, level of moisture content, and the population of microflora present determine the type of microorganism that dominates the entire forage. As lactic acid bacteria increase in number and utilize water soluble carbohydrates to produce lactic acid, the pH of the silage drops below the critical point inhibiting the growth of other spoilage microorganisms. At this stage, most acid tolerant Clostridia will be inhibited [51].

### 5.3. Fermentation or Stable Phase

Dramatic decrease in pH hinders the growth of Clostridia and enterobacteria. During this period, dry matter plays a significant role in fermentation processes. As the moisture content increases (greater than 70%), growth of lactic acid bacteria is hampered; thereby the rate and extent of fermentation are reduced [8]. This phase is the longest in ensiling process and it continues until the growth of microorganisms is completely inhibited by high acid level (low pH) [52]. At this point, the forage is in a preserved state. It should be noted that undesirable bacteria tend to thrive and such results in excessive protein degradation, production of toxic substances, and loss of dry matter [53]. Therefore, wilting forage above 30–35% dry matter prior to ensiling can eliminate undesirable bacterial.

### 5.4. Feedout Phase

During this phase, good silage will remain stable and unchanged in composition. However, pH value alone cannot indicate good quality of the silage or type of fermentation process. The end product of silage fermentation is often measured or monitored using some indicators in order to assess the silage quality and compositions as indicated in Tables 2 and 3.

## 6. The Silage Microflora

The silage microflora plays an active role in the successful outcome of fermentation processes. These microfloras are divided into two main groups: desirable and nondesirable. The desirable microfloras are lactic acid bacteria while the nondesirable microfloras are categorized based on their roles in silage spoilage. Aerobic microfloras are involved in anaerobic spoilage (e.g., enterobacteria and Clostridia) while anaerobic ones are involved in aerobic spoilage (e.g., moulds and yeasts). Both aerobic and anaerobic microflora spoilage does have effects not only on silage product but also on the animals that consume it [14].

## 7. Lactic Acid Bacteria (LAB)

Lactic acid bacteria (LAB) are a clade of gram-positive bacteria, nonrespiring, nonspore forming, cocci or rods shaped, acid tolerant, and epiphytic in nature. Lactic acid bacteria associated with silage belong to the genera* Leuconostoc*,* Lactobacillus*,* Enterococcus*, and* Pediococcus* [48]. Water soluble carbohydrate and dry matter content of silage enhance the growth and multiplication of LAB [14]. Most LAB strive best at temperatures between 20 and 40 degrees with an optimum temperature of 30 degrees. The whole base of LAB in silage is centralized on their ability to reduce the pH value which can be reduced to 4 or 5 depending on the fodder crop [14]. All LAB naturally respond to aerobic condition but some, however, give preference to anaerobic condition. Lactic acid bacteria are divided into two groups based on sugar metabolism: homofermenters and heterofermenters. Some of the most common homofermenters and heterofermenters used in silage inoculants include the following:

- *Lactobacillus plantarum* (homofermenters) are used to produce rapid amount of lactic acid and are relatively tolerant to an acidic condition.
- *Enterococcus faecium* (homofermenters) are used for fast production of lactic acid and are faster in growth rate than* Lactobacillus*.
- * Pediococcus acidilactici* (homofermenters) tolerate a wide range of osmotic pressures (osmotolerance), show a good growth rate at cooler temperature, produce lactic acid with rapidity, and grow at faster rate than* Lactobacillus*.
- *Lactobacillus buchneri* (heterofermenters) are used to produce antifungal compounds; the end products are lactic acid, acetic acid, propanediol, and carbon dioxide.

## 8. Enterobacteria

Enterobacteriaceae belong to the group of epiphytic microfloras found on most forage crops. Among these groups,* Rahnella aquatilis *and* Erwinia herbicola* dominate the fresh crop but are rapidly replaced by* Serratia fonticola*,* Escherichia coli,* and* Hafnia alvei* after ensiling [54].* Escherichia coli* have been found to be the most important species in this group from the perspective of health risk [14]. Enterobacteria have weak proteolytic activities and are able to decarboxylate and deaminate some amino acids. These activities contribute to the production of ammonia [55] and biogenic amines in silage [14]. Biogenic amines are toxic compounds that have an adverse effect on silage feed intake and palatability in ruminants [56]. Another special characteristic of enterobacteria is their ability to reduce nitrate (NO_3_^−^) to nitrite (NO_2_^−^), nitrite to nitrous oxide (N_2_O), and ammonia [57]. During ensiling, the linkage of air causes the nitric oxide to oxidize into a mixture of gaseous nitrogen oxides (NO_2_, N_2_O_3_, and N_2_O_4_). Finally, nitrogen dioxide and nitric oxide react with water to give nitric acid (HNO_3_) and nitrous acid (HNO_2_). These gases and acids have been reported to cause respiratory irritation and silo filler's disease with symptoms of pneumonia in human beings [58]. However, the metabolic activity of the enterobacteria is readily inhibited during the conservation process either by anaerobiosis or by acidification through increase in lactic acid by the LAB at pH of 4.5 to 5.0.

## 9. Clostridia

The genus* Clostridium* is present in silage as contaminants that cause anaerobic spoilage as well as negative impact on the feeding value. Many of its species are capable of saccharolytic and proteolytic (breaking down carbohydrate and protein). The most common species of* Clostridium* associated with silage include* C. bifermentans*,* C. sporogenes*,* C. butyricum*, and* C. tyrobutyricum* [48]. The first two species are extremely proteolytic while the latter two are mildly proteolytic. Among these species,* C. tyrobutyricum* is the most studied Clostridia in silage as a result of acid tolerant properties to the extent that pH of 4.0 may not inhibit its growth [14]. There are two main types of Clostridia spp. associated with silage fermentation spoilage, namely, saccharolytic types and proteolytic (putrefactive) types. The saccharolytic types are characterized by their ability to ferment sugar and lactic acid to produce hydrogen, carbon dioxide, and butyric acid. They include* C. tyrobutyricum*. It has also been reported to be responsible for a cheese defect called late blowing or hard cheese production [14, 59] resulting from faecal contamination of udder with its spores. Silage that undergoes this type of fermentation is characterized by increase in pH and butyric acid. Since this acid is weaker than lactate, it favours the growth of other putrefactive microorganisms including proteolytic Clostridia. The proteolytic (putrefactive) types attack amino acids and protein. Their activities involved deamination (where ammonia is liberated from amino acid), decarboxylation (formation of amines), and oxidation/reduction (where amino acid is oxidized or reduced forming organic residues). Silage that undergoes clostridial fermentation is characterized by high pH and high content of ammonia, butyric acid, and amines. However, rapid decline in pH inhibits its growth and water activities (WA) since Clostridia are susceptible to low water availability resulting from wilting of plant materials prior to ensiling [60]. The amount of ammonia (NH_3_) present in the silo is a good indicator of the amount of proteolytic clostridial activity as it is only produced by other silage microorganisms in very small amounts.

## 10. Yeasts

Acid tolerant species of yeasts associated with silage belong to the genera* Torulopsis*,* Hansenula*,* Saccharomyces*, and* Candida* [61]. Silage with excess yeast value of 10^5^ cfu g^−1^ is set to undergo yeast spoilage [62]. During ensilage, yeasts ferment sugar to carbon dioxide and ethanol. However, the presence of ethanol in silage increases loss of dry matter [63] and also affects the milk taste [64]. Yeasts are one of the most important groups of microfloras responsible for initiating aerobic spoilage during ensilage [61]. Its growth can be checkmated by the pH level, anaerobiosis, and the concentrations of organic acids [23].

## 11. Moulds

Moulds are eukaryotic microorganisms that depend on aerobic respiration for their metabolic activities. Some species of mould (*Byssochlamys nivea*,* Penicillium roqueforti*, and* Aspergillus fumigatus*) can survive in very low volume of oxygen [59]. Their growth is restricted to the surface of silage and their presence in silage is as a result of poor compaction and sealing. The most common genera of moulds associated with silage are* Aspergillus*,* Mucor*,* Penicillium*,* Arthrinium*,* Absidia*,* Fusarium*,* Byssochlamys*,* Monascus*,* Scopulariopsis*,* Trichoderma*, and* Geotrichum* [65]. Moulds reduce palatability and feeding value of silage. They also have detrimental effects on animal health because of mycotoxin production [66]. The effects of mycotoxin in silage could be minor problems like indigestibility, reduction in immunity system, and fertility problem or major and complicated problems like abortion and kidney and liver failure [67]. Proper sealing and compacting can prevent entry of air and ensure pH reduction. With these precautions, growth of moulds could be limited or prevented and initiation of aerobic spoilage is stamped out.

## 12. Silage Additives

Silage fermentation process is a unique procedure that can be affected by different factors. Different silage additives are used to increase nutrient and energy recovery, reduce fermentation losses, promote rapid fermentation, and improve animal performances. It is worth noting that silage additives will not change poor quality forage into good one but rather improve good quality forage to become excellent [26]. Silage additives should be safe to handle, improve hygienic quality, increase nutritive value, limit secondary fermentation, reduce dry matter (DM) losses, reduce aerobic deterioration during feedout, increase animal production, give high returns to farmers, and be cost effective [26]. Based on available literatures, additives used in silage process are classified into three major categories according to their mode of actions (Table 4).

- Fermentation stimulants: these promote production of desirable lactic acid to lower the pH of silage [15, 31].
- Fermentation inhibitors: these directly increase the acidic level on the silage and inhibit the growth of unwanted microorganisms [68].
- There are nutrient additives or nonprotein nitrogen, for example, urea and ammonia [6, 69].

## 13. Fermentation Stimulants 

In order to improve fermentation quality of rice straw silage, some additives are used. For example, application of LAB (Chikuso-1) to round baled whole rice crop could improve the silage quality with lower pH value, low butyric acid (BA) and low ammonia nitrogen (NH_3_–N) concentrations, and higher lactic acid (LA) and crude protein (CP) concentrations [9]. Also, inoculation with NLRI401 (*Lactobacillus plantarum*) could lower the pH of the silage and butyric acid (BA) concentration and increase lactic acid (LA), acetic acid (AA), and crude protein (CP) concentrations in the rice straw silage [10]. Additionally, inoculation with LAB could also decrease neutral detergent fiber (NDF) and acid detergent fiber (ADF) concentrations and increase digestibility of dry matter (DM) and NDF of rice straw silage [70]. In summary, fermentation stimulants used in silage additives promote the desired lactic acid fermentation and subsequently improve silage preservation by either increasing the population of desired lactic acid bacteria or providing additional sugars used by silage bacteria.

## 14. Microbial Inoculation Stimulants

The primary motive of microbial inoculation on silage is to add fast growing lactic acid bacteria (LAB) to dominate the entire fermentation process. This encourages production of more lactic acid to inhibit the growth of other silage spoilage organisms and preserve forage mass. The most commonly used micros in silage are lactic acid bacteria like* Enterococcus*,* Pediococcus*, and* Lactobacillus* species. Theoretically, inoculation of LAB is to produce lactic acid to increase the acidity level of the silage resulting in low dry matter losses as compared with use of naturally occurring bacteria which results in production of acetic acid, lactic acid, butyric acid, ammonia nitrogen, ethanol, carbon dioxide, and mannitol. LAB inoculation is more effective if the population of naturally occurring microbes is less than 10,000 organisms per gram of fresh sample [71]. This is because naturally occurring microbes compete with inoculants. The microbial inoculant must be present in high amount in order to dominate the entire forage. The recommended rate for* Lactobacillus plantarum* inoculum is 100,000 colony forming units (1 × 10^5^) per gram of wet forage [71]. Chlorinated water should be avoided especially if it exceeds 1.5 to 2 parts per million because it reduces the effectiveness of the bacteria [72]. In order to understand the effect of inoculant additives on the performance of the animal, Table 5 describes the possible reason for these improvements on the performance of the animals.

## 15. Substrate Suppliers (Enzymes Additives Stimulant)

Enzymes are naturally occurring biological catalyst that speed up the rate of biochemical reactions by lowering activation energy of the reactants. Enzyme additives used in silage production have two major functions [17]. Firstly, they release free sugars from forage plant during ensiling to be used by fermentative microbes in producing acetate and lactate and thus preserving the forage by lowering the pH. The second function is degradation of cell wall to lower the total fiber contents of the ensiling materials. All of these improve the digestibility and intake of organic matter. Furthermore, there is promotion of breaking down complex feed molecules into smaller organic molecules such as glucose and amino acids which could be digested by ruminant animals. Enzymatic hydrolysis is used to convert rice straw to sugars [73]. Colombatto et al. [74] have found that enzymes enhance fermentation of cellulose and xylan by a combination of pre- and postincubation actions. Commonly used enzyme-based silage additives include hemicellulases, cellulases, xylanases, pectinases, and amylases. They are used for degrading fiber portion of forage. The preferred hemicellulose enzyme is used for improving overall fiber digestibility. Hemicellulose is composed of various components including a backbone of xylans and arabinose side chains. The arabinose side chains are typically more digestible than the xylan backbone. As a result, enzyme mixtures that focus on breaking down the xylan backbone of the hemicellulose have a better potential to increase sugar content of the forage and improve digestibility. Xylan-rich cell walls of rice straw contain a significant amount of lignin that is resistant to enzymatic hydrolysis. Therefore, rice straw requires severe pretreatments like alkali digestion, acid hydrolysis, steaming, and radiation before the polysaccharides can be enzymatically hydrolysed to monomer sugars. Studies on fibrolytic enzymes have shown that they cannot significantly increase the degradability of rice straw due to limited ability of these enzymes to break down the esterified bonds within lignin-carbohydrate complexes [75]. However, some reports have shown increase in degradability and in vitro fermentation characteristics when fibrolytic enzymes are combined with other pretreatments [31]. Various combinations containing both enzymes and lactic acid bacteria have also been developed. In theory, these additives complement each other by utilizing additional substrate provided by the enzymes during the fermentation process. This was demonstrated by Liu et al. [75] who treated rice straw with cellulase from* Penicillium funiculosum *in combination with steam pretreatment. In the same vein, Wang et al. [76] treated rice straw with multiple enzymes (xylanase, *β*-glucanase, carboxymethyl cellulase, and amylase) in combination with NaOH. Finally, Eun et al. [77] treated rice straw with xylanase or cellulase in combination with ammonia. Combination of fibrolytic enzymes with pretreatments is expected to have a synergistic effect on the nutritive improvement of rice straw. Enzyme additives are used to break down complex carbohydrates in the forage to release simple sugars (water soluble carbohydrates) that can be utilized by lactic acid bacteria (LAB) to improve silage fermentation quality. The sugars produced, just like those in molasses, are easier for lactic acid bacteria to use as a food source to grow. Enzyme additives have potential to increase fiber digestion and productivity of ruminants. It also improves in vitro fiber digestion and nutritive value of both low quality forage and high quality forage [78]. Enzyme additives used in silage include

- hemicellulase which breaks down the hemicellulose fiber to yield xylose, xylans, and arabinose,
- cellulase which breaks down cellulose fiber into glucose, maltose, and limited dextrins,
- amylase which breaks down starches into glucose and maltose,
- pectinase which breaks down pectin fiber.

Responses observed in enzyme additives have been reported in many studies. For instance, Kaiser [72] conducted a review on available evidence and found that there was reduction of 50–60% in neutral detergent fiber (NDF) and acid detergent fiber (ADF) of experiments studied. He also found that silage fermentation was improved in less than 50% of the experiments and aerobic stability was unchanged in two-thirds of the studies while DM losses during storage were unchanged in more than 70% of researches available for his study. Some studies have shown improvement in animal performance (i.e., greater milk yield) [79], while others have shown no benefit from feeding enzyme treated silage to ruminant animals [80]. Circumstances surrounding the issue of enzymes as effective additives in forage improvement could not be understood until the observed inconsistencies in different experiments are studied to determine the contribution of pH, forage types, moisture content, temperature, incubation time, kind of forage, its characteristics, and type of processing of access of enzymes to target cell wall tissues. However, enzyme additives vary in effectiveness (efficiency of fiber-degrading) depending upon conditions set to achieve maximum activities. The following are the factors to be considered.

- *Enzyme Type and Application Rate*. An enzyme's effectiveness increases with the quantity and quality (enzyme type) applied.
- *Lactic Acid Bacteria (LAB)*. Population of lactic acid bacteria plays a significant role in determining the effectiveness of enzyme treated rice straw.
- *Time*. Cellulases and hemicellulases are active over a prolonged period but their activity is related to pH.
- *pH*. Cellulase activity is optimum at pH of 4.5. This occurs naturally at the later stages of fermentation process when optimum activity is reached. Nevertheless, optimum pH varies with cellulase sources.
- *Varieties*. Differences in the fermentation quality of silage among rice varieties depend mainly on water soluble carbohydrate concentration in the straw. Based on this, Vadiveloo [81] suggested that rice varieties with superior straw characteristics should be cultivated to improve utilization of the residue. In the same vein, hemicellulose and cellulose contents of rice straw differ with varieties and cultivation seasons [69].
- *Temperature*. Enzyme activity increases with temperature though excessive heating in silage stack or bale reduces enzyme activity. Cellulases are usually active within 20–50°C temperature range with optimum activity at the upper end of this range.

## 16. Molasses (Stimulant Sugar)

Sugar or sugar-rich materials (molasses) are commonly used as an effective additive for ensiling crops that have low WSC. To improve fermentation quality, molasses additives are used as stimulants to increase the supply of fermentable carbohydrate to enhance the growth of LAB [15]. Molasses is a by-product of starch, sugarcane, citrus, hemicellulose, and sugar beet. It is made of 45 to 50% of sucrose containing 79% soluble carbohydrates. In numerous experiments, molasses additives have been proved to improve fermentation quality in terms of fast acidification which prevents growth of Clostridia. Molasses additives also reduce ammonia nitrogen (NH_3_–N), decrease organic matter losses, lower the level of volatile basic nitrogen, and reduce pH of the silage materials [11]. Although molasses treatment increases silage quality and dry matter intake, it does not improve digestibility and animal performance [82]. The relative high viscosity of molasses makes it more difficult to apply as additives than other by-products like pineapple and citrus pulp. Therefore, it is often mixed with water at ratio 1 : 1 to ease application. However, adding sugar alone might induce proliferation of undesirable microorganisms which results in dry matter losses [11]. To forestall this problem, it is often applied along with bacterial inoculation [15] or enzyme additives [31].

## 17. Inhibitors (Acids)

Acids are used during ensiling to initiate rapid drop in pH to inhibit growth of undesirable microbes. They also reduce fermentation losses of carbohydrate and protein [26]. They also reduce heat losses and enzyme activities and prevent further plant respiration during the first phase of ensiling due to their ability to sink plant materials and consolidate them [83]. Addition of acid increases silage influent which can be potentially harmful to human beings, animals, and machinery. Therefore, regulation of silage moisture level reduces the magnitude of influent. In the same vein, addition of calcium carbonate can be used to adjust the pH of silage during ensiling. Liu and Guo [84], however, proposed the application rate of the commonly used acid additives in silage as follows:

- Formic acid applied at 3 kg/ton or 3 kg/ton formic acid.
- Propionic acid at the rate of 1 litre/m^2^ or 1 litre/m^2^ propionic acid.
- Acetic acid at 5–20 kg/ton or 5–20 kg/ton acetic acid.

Formic acid does inhibit growth of bacteria through acidification. This characteristic is also present in propionic acid [85]. In contrast, sulphuric acid, hydrochloric acid, and mineral acids only reduce pH with no antimicrobial characteristics [85]. Apart from bacteriostatic properties of formic acid, it also prevents protein degradation during ensiling [86]. This is achieved through rapid decline in pH and binding of formaldehyde to the nitrogenous components of the silage [48]. According to McDonald [48], yeast is particularly tolerant to formic acid due to increase in yeast count and restriction in fermentation. These lead to poor fermentation which results in higher content of residual water soluble carbohydrate (WSC). Application of formic acid at low levels initiates the growth of Clostridia while high level application causes reduction in feed intake and digestibility [86]. Therefore, understanding the application rate is a prerequisite for ensiling.

Propionic acid is a short chain fatty acid having the greatest antifungal activities [87]. Although it is weaker than formic acid, it is very effective in reducing moulds and yeast growths which are responsible for aerobic deterioration during ensiling. In the past, propionic acid is often applied in higher quantity (1 to 2% of the dry matter) to improve aerobic stability but it often restricted fermentation processes [88]. Application of propionic acid depends on the moisture content of ensiling materials, formulation, and other preservatives [87].

In spite of the positive impacts of acids and most especially the organic ones, they are difficult to handle because of their corrosiveness to farm machinery and high health risks except with necessary precautions. This has led to using alternatives to acids in the form of acid salts in some commercial products. The efficacy of acid salts is ingrained in their inhibitory effects on Clostridia [86]. Their activities are similar to organic acids if active ingredients are applied at the same rate as that of organic acid. According to Bolsen et al. [85], application of acid salts with lactic acid bacteria results in reduced fermentation losses, improved aerobic stability, and lower pH compared with LAB inoculation alone. However, there is paucity of information on application of this treatment.

## 18. Nutrient Additives or Nonprotein Nitrogen 

Chemical pretreatment is another method of improving the nutritive value of rice straw. These pretreatments are designed to increase feed intake and more importantly digestibility. This method involves treating straws with alkaline solutions such as ammonia, sodium hydroxide, and calcium hydroxide [89]. It is basically a delignification process through disruption of lignin structure by separating the linkages between carbohydrates and lignin for solubilisation of significant amount of hemicellulose [90, 91]. Moreover, it physically makes structural fibers swollen and thereby increases the number of accessible sites of microbial attachments on the surface of the particles to have higher degradability and better intake by animals [6].

Urea treatment is a conventional method of increasing the nitrogen level of ensiling materials through increasing the protein content and digestibility [92]. Shen et al. [7] have reported degradation of cellulose and hemicellulose with decrease in organic and dry matter losses when straw was treated with 5% urea. Urea is the most popularly used pretreatment of rice straws because it is nonhazardous and serves as a delignifying agent through ammonification [93]. In addition, urea treatment causes the removal of silica polymerized cuticle waxes from the surfaces of the leaf sheath and leaf blade [7]. It also exposes the underlying tissues of straw to bacterial colonization [94]. Furthermore, it reduces hemicellulose contents of the straws and increases their extractable biogenic silica contents [69]. In the same vein, it increases feed intake, animal production, and digestibility in dairy cows [93] and goats [95].

Addition of ammonia increases the pH of silage to 8 or 9 [32]. With this high pH and ammonia effect on silage, the growth of mould and yeast population is inhibited and consequently increases aerobic stability of the silage materials. Addition of ammonia also inhibits plant proteases which reduces the rate of protein degradation during ensiling. Application of ammonia reduces the number of LAB which causes delay in fermentation process. It also causes breakage between hemicellulose and other cell components to extent and rate of digestibility in the rumen. The reasons adduced to improvement of ammoniation of straw include ability of ammonia to form ammonia cellulose complex and reduce cellulose crystallinity [96]. Other reasons for improvement of straw ammoniation are collapse of vascular bundle cells in rice straw treated with ammonia [97] and separation of ground parenchyma and inner cuticular surfaces when straw was ammoniated [98]. Furthermore, there are dissolution of parts of hemicellulose and cleavage of ester bonds in uronic acids with loss of acetyl groups to release acetic (phenolic acids), which may be the reason for decreases in NDF and ADF [99]. Goto and Yokoe [100] have established that a more rapid fragmentation of ingested treated material would increase the surface area available for microbial attack and the rate of breakdown would increase the rate of passage of treated straw through the digestive tract. Ammonia additive results in increase in crude protein and prolonged aerobic stability, lowers mould population and temperature while ensiling, and increases protein degradation in the silo. When using ammonia additive, caution must be taken because excess ammonia may result in poor fermentation (because of a prolonged buffering effect) and low animal performance. Since ammonia is corrosive to zinc, copper, and brass, materials made of zinc, copper, and brass should be avoided while ensiling ammonia treated forage. Aqueous NH_3_ is more technically difficult to handle and may expose the handler to health hazards while urea treatment does not pose such problems. Both urea and ammonia additives prolong fermentation because of their buffering effects which result in greater total acid production. Their buffering effect on silage is a problem because it leads to low WSC of rice straw. Therefore, it is recommended that when using these additives, molasses or other sugar relatives should be added for better quality [26].

## 19. Factors Affecting Silage Dry Matter Intake

Nutritive value of forage silage is mainly determined by feed intake and digestibility. The variation observed in ruminant performance is closely related to the amount of feed intake rather than net energy or diet digestibility [101]. In order to overcome the variation in animal performance, Huhtanen et al. [102] presented relative silage dry matter intake (SDMI) approach for assessing feed intake. Potential silage dry matter intake is determined by silage fermentation quality, maturity stage, whole crop silage, protein supplement, and wilting.

### 19.1. Fermentation Quality

Ammonia N together with some other end products of silage proteolysis has consistently decreased silage DM intake [102], although it has been suggested that decrease in SDMI is not due to ammonia N per se [103], and it may be an indirect effect due to correlation of ammonia N with some other variables such as 2-butanol, 1-butanol, and butyric acid. Rook and Gill [104] suggested, based on high correlation between VFA and ammonia, that VFA was the causative factor. According to Buchanan-Smith and Phillip [105], soluble constituents in silage were reported to inhibit feed intake; however, no single component was reported to be sole causes of this inhibition. Instead emphasis was put on the importance of protein quality. Protein degradation products such as biogenic amines have been suggested to be responsible for reduced silage intake with increasing proteolysis in sheep [106]; however, after an adaptation period intake returned to the initial level.

### 19.2. Maturity

During maturation phase, accumulation of tissue and secondary thickening are more in the stem as compared with the leaf material, therefore, leading to higher concentration of lignin, cellulose, and xylan in the cell wall [107]. Plant cell contents are virtually and completely digestible while the cell wall concentration is not fully digestible making it the key important factors for forage intake. Forage digestibility and intake are largely subjected to the fiber content and more precisely digestible neutral detergent fiber (NDF). This potential digestible NDF is defined as the fraction available for microbial digestion which disappears after a long incubation period and the rest is referred to as indigestible component of NDF (iNDF). Minson [108] reported that, under certain condition, NDF is a reliable predictor of voluntary dry matter (DM) intake. The relationship between the feed intake and NDF is not a linear relationship rather complex one because the quality and amount of NDF can either limit or enhance intake [108]. The intake of DM decreases at high concentration of NDF range between 22.2% and 45.8% while there is increase in feed intake at lower concentration of NDF concentration between 7.5% and 35.5% [109]. Digestibility of high content of NDF in diet is greatly influenced by iNDF which is the main limiting factor of forage intake [110]. The lack of digestibility in the iNDF fraction of forage is attributable to the cross-linking between cell wall lignin and hemicellulose [110]. A higher iNDF intake limits a ruminant's ability to consume sufficient forage to meet nutrient requirements. The indigestible portion is removed from the rumen by passage only and will accumulate in the rumen relative to the potentially digestible portion, therefore having a longer rumen retention time. A longer retention time in the rumen results in a lower intake [110].

## 20. Conclusion

Success in production of quality silage depends on two main factors. The first one is the nature of ensiling materials which determines the microbial population, buffer capacity, dry matter content, water soluble sugar, and chemical composition. The second factor is the mechanism or strategy of pretreatment. Although different biological, chemical, and physical treatments of rice straw have been used to improve digestibility and feed intake of ruminant animals, the practical use of these treatments is only restricted to significant cost effectiveness and safety. A good method of silage fermentation increases the feeding value of silage, improves its quality, and betters animal performance. The use of additives in association with good management practices and safety could improve the quality of silage. Methods shown to improve silage feeding value include effective wilting and rapid acidification, either by direct acidification or by use of inoculants. Relevant criteria for choosing additives are chosen by considering cost effectiveness, improvement of dry matter recovery, aerobic stability, and animal performance.

## Figures and Tables

**Table 1:** Abundance of rice straw across the continents of the world in 2014.

Continent	Rice production (million tonnes)	Rice straw (million tonnes)^*∗*^
Oceania (Australia)	0.9	0.72
Europe	4.1	3.28
North America	12.8	10.24
South America	25.4	20.32
Africa	27.6	22.08
Asia	673.6	538.88

Total	744.4	595.52

^*∗*^The amount of rice straw generated (dry ton/year) is 0.8 tonnes per tonne of rice produced (ton/year).

*Source*: [12].

**Table 2:** Chemical properties of silage, characteristics, and interpretation.

Silage characteristics		Interpretation	Reference
Chemical composition	Lactic acid	High concentration of lactic acid lowers pH and has positive effects on silage by inhibiting the growth and activities of undesirable bacteria	[14]
	Butyric acid	A high concentration of butyric acid indicates degradation of protein content and large amount of dry matter loss and energy wastage	[15, 16]
	Acetic acid	High concentration of acetic acid shows the activity heterofermentative LAB thereby increasing aerobic stability; it also possesses antifungal activity able to reduce the development of undesirable spoilage organisms in ensiled mass and improving the fermentation quality of silages	[17]
	Ethanol	Higher ethanol concentration has a negative impact on the silage quality and indicates that the silage has undergone the activity heterofermenter LAB and suffered dry matter loss	[17]
	Acid detergent insoluble nitrogen	High concentration of acid detergent insoluble nitrogen (ADIN) indicates high level of heat damage on protein and low energy content	[18]
	Ammonia	High ammonia concentration shows that there is excessive breakdown of protein during fermentation	[16]

**Table 3:** Physical properties of silage, characteristics, and interpretations.

Silage characteristics		Interpretation	Reference
Physical appearance and texture	Rotten silage or presence of mould	Indication of air leakage which results in DM loss and in turn decline in silage quality	[19]
	Very wet with discharge seeping from stack	The ensiling is low in DM; this results in poor fermentation which leads to significant losses in silage quantity and quality	[20]
	Very dry and even breakable	The ensiling is too high in DM content; this results from overheating during storage and leads to protein degradation and reduction in metabolizable energy	[21]

Colour	Light amber brown	This is due to late harvesting or low DM content; the bottom layer of wet silage can be yellowish with fruity smell	[22]
	Brown/dark brown	This occurs as a result of overheating or inadequate compaction or delayed sealing or aerobic spoilage which leads to low digestibility and protein degradation	

Aroma	Alcoholic, fruity, or sweet smell	The silage product is unstable during feedout often as a result of yeast fermentation resulting in high level of ethanol	[22]
	Rotten aroma	The silage is dominated by Clostridia bacteria which increase the level of butyric acid resulting from protein degradation	

**Table 4:** List of available silage additives used for ensiling.

Additives	Activities	Examples
Stimulants	Microbial inoculation	Lactic acid forming bacteria (homofermentase and heterofermentase)
	Enzymes	Cellulases, amylases, hemicellulases, pectinase, and proteases
	Sugar	Glucose, sucrose, molasses, citrus pulp, pineapple pulp, and sugar beet pulp

Fermentation inhibitors	Acid and organic acid salt	Aerobic (e.g., propionic acid, sulphates, caproic acid, sorbic acid, propionates, and acetic acid) and anaerobic (e.g., formic acid, mineral acids, lactic acid, benzoic acid, acrylic acid, citric acid, and sorbic acid)
	Other chemical inhibitors	Sodium nitrite, formaldehyde, and sodium metabisulphite

Nutrients	Nonprotein nitrogen	Urea and ammonia

*Source*: [23–25].

**Table 5:** Effects of silage additives on fermentation and possible improvement in animal performance.

Effect(s) of silage additive	Results and possible effects on animal performance	Reason(s) for effect(s)	Reference
Lower pH	Inhibition of undesirable microbes; improved protein preservation and nitrogen metabolism	Dominance of homofermentative lactic acid bacteria	[9]

Inhibition of fermentation, for example, high levels of acid addition or other fermentation inhibitors	Improved intake due to reduction in overall fermentation of end-products and reduced acidity	Inhibition of microbial growth	[26]

High lactic acid: VFA ratio and low acetic acid concentration	Low acetate may result in increased DM intake and improved rumen microbial fermentation and palatability	Dominance of homofermentative lactic acid bacteria	[11]

High propionate concentration	Improved aerobic stability leading to less spoilage; better feed intake and less mycotoxin formation	Direct addition or microbial production	[27]

Low butyric acid	Improved feed intake	Lower pH which leads to inhibition of Clostridia	[28]

Low ammonia N, free amino acids, and amine concentrations	Improved nitrogen metabolism and feed intake	Dominance of homofermentative lactic acid bacteria causing rapid drop in pH and inhibition of plant proteases	[29]

High concentrations of peptides	Possible stimulation of microbial protein production	Rapid drop in pH and dominance of homofermentative lactic acid bacteria	[30]

Low concentrations of ethanol	Improved aerobic stability and possible improvement in feed intake	Inhibition of yeasts which are primarily responsible for aerobic spoilage	[9]

Low fiber contents	Improved nutrient utilization	Partial digestion of fiber by enzymes	[29]

Increased water soluble carbohydrates	Low aerobic stability and feed intake	Partial digestion of fiber by enzymes	[31]

Improved nutrient digestion, for example, fiber or starch	Change in physical or chemical structure of fiber, better nutrient/energy utilization, faster rate of digestion, and improved feed intake	Partial digestion of fiber by enzymes and unknown effects of lactic acid bacteria	[32]

Improved aerobic stability (less heating and moulding)	No mycotoxins; improved nutrient intake	Inhibition of yeasts which are primarily responsible for aerobic spoilage	[30]

Increase in lactic acid bacteria	Probiotic effect or other unknown effects (e.g., bacteriocins) resulting in improved feed intake or conversion	Addition of inoculants	[11]
